# Solitary Bone Cyst in the Lumbar Spine: Case Report and Literature Review

**DOI:** 10.1155/2024/9975362

**Published:** 2024-06-06

**Authors:** José Ramírez-Villaescusa, David Ruiz-Picazo, Ana Verdejo-González, Adriana Canosa-Fernández, Pedro Torres-Lozano, Gracia Guerrero-Álvarez

**Affiliations:** ^1^ Department of Orthopaedic Spine Surgery Unit Complejo Hospitalario Universitario de Albacete, Albacete, Spain; ^2^ Department of Pathology Complejo Hospitalario Universitario de Albacete, Albacete, Spain

## Abstract

**Introduction:**

To describe a rare case of solitary bone cyst in the vertebral body of the lumbar vertebra in an adult patient. The solitary bone cyst is defined as a cystic lesion with liquid content. Few cases have been described in the vertebral location without preference for the posterior arch or vertebral body. Most have been treated with resection, curettage, and/or grafting. No case described to date has been treated with polymethylmetacrylate (PMMA) injection in the vertebral location. *Case Presentation*. A 50-year-old male patient was consulted for lumbar pain with no traumatic history and no neurologic deficit. The radiological study showed lumbar arthrodesis with L2-L4 instrumentation due to an L3 fracture twenty years earlier. Computed tomography (CT) scan showed a lytic lesion occupying practically the entire vertebral body of L5, with incomplete septum and sclerotic edge, without cortical rupture. The previous steel instrumentation was removed, to avoid the presence of artifacts when performing the magnetic resonance (MR), and a biopsy of L5 vertebra was performed via transpedicular in the same act. The MR study findings and biopsy were compatible with the simple bone cyst. Finally, a new intervention was performed by filling the lesion with PMMA. Follow-up at 5 years was satisfactory without lumbar pain as well as the radiological study and with a return to previous activity.

**Conclusions:**

The spinal location of the simple bone cyst is extremely infrequent. Its diagnosis excludes other lesions and is made by imaging studies and biopsy. Treatment can be performed by excision, curettage, or filling with graft or as in this case, with PMMA.

## 1. Introduction

The simple or solitary bone cyst (SBC) is a benign pseudotumoral lesion of liquid content, generally single and unicameral, although occasionally segmented [1]. It is more frequent in the second decade of life and predominantly 2/3 : 1 in males. It is located in the proximal metaphysis of the humerus and femur in 80% of cases, infrequent in other locations and exceptional in the spine with very few cases described in this location [2].

SBC is described as a pseudotumoral lesion of dysplastic or reactive origin due to bone resorption by blockage of the venous circulation with an increase of proteins in the cyst contents [3] or to the persistence of synovial debris [4]. The diagnosis is made incidentally as a finding in a radiological study or due to spontaneous or post-traumatic pain after a fracture. In the infrequent vertebral location, it may cause pain of a mechanical nature or radicular pain due to cyst rupture in the pedicular location [5].

The radiological study allows diagnosis in most cases. The lesion is single, elongated, radiolucent, discreetly expansive, and thinning the contiguous cortex. The so-called “fallen leaf sign” due to an intracystic free cortical fragment is a characteristic. A computed tomography (CT) can be useful in vertebral localization to evaluate the cystic walls, the presence of septa, and the risk of fracture due to thinning of the cystic walls. Through magnetic resonance imaging (MR), due to the liquid content, the lesion shows a normointensity or hypointensity aspect in T1 sequences and homogeneous hyperintensity in T2-weighted sequences [1]. Biopsy may be necessary to rule out other primary or metastatic malignant lesions. Aneurysmal bone cyst, giant cellular tumors, and primary or metastatic lesions should be excluded.

In vertebral location, previous studies show the different treatment options such as (1) aspirate with corticosteroid injection [6] has been described for smaller lesions in the vertebral body; (2) simple resection [7–9], curettage [10–15], or resection with curettage [8, 11, 15]; (3) other authors have described curettage and filling with autologous graft [2, 16–22] or filling with hydroxyapatite [13]; (4) resection with arthrodesis due to the location and possible instability after resection [5, 10, 23, 24]. No case described to date has been treated with polymethylmethacrylate (PMMA) injection in vertebral location.

We present a rare case in an adult patient of SBC in an adult patient located in the vertebral body of the lumbar vertebra L5 and treated with PMMA.

## 2. Clinical Case

A 50-year-old male is referred for low back pain for months of evolution with no traumatic history. He has a history of previous surgery after a traumatic episode due to a vertebral fracture 20 years earlier. Clinical examination shows lumbosacral pain without neurologic involvement in the lower limbs. Laboratory studies showed no remarkable alterations.

**Figure 1:**
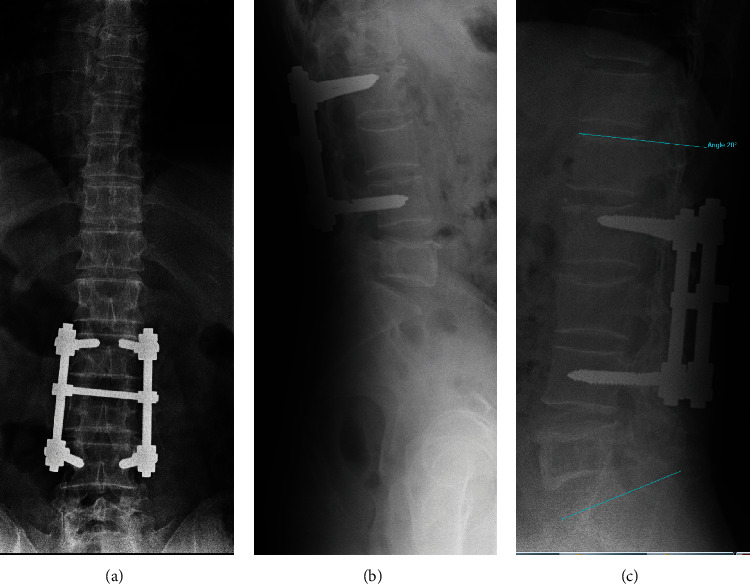
X-ray anteroposterior (a) and lateral preoperative views (b/c) showing previous instrumentation post-fracture intervened years before with lumbar lordosis 28°.

**Figure 2:**
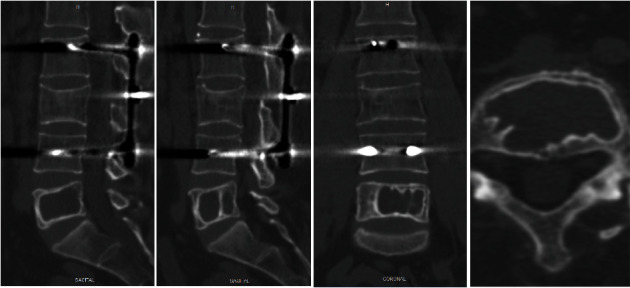
CT sagittal, coronal, and axial images. Sagittal view shows incomplete septum at L5 vertebral body.

The radiological study of the lumbosacral spine in standing position showed vertebral instrumentation at L2 and L4 levels without loss of height of the L3 vertebral body. No traumatic vertebral lesions or implant ruptures were observed. The lumbar lordosis L1-S1 was 23°. In the lateral view, an image of geographic aspect was observed in the vertebral body of L5 without vertebral insufflation or loss of vertebral height (Figure 1). The computed tomography (CT) study showed a lytic lesion that occupied and replaced practically the entire vertebral body of L5, with incomplete septum and sclerotic edge, with cortical integrity and no calcifications inside (Figure 2).

Due to the characteristics of the lesion and the presence of steel implants, it was decided to remove the previous instrumentation, perform a biopsy, and complete the imaging study by magnetic resonance (MR). Under general anaesthesia, the instrumentation was removed without incident, and a sample was taken through the left transpedicular route of the contents of the vertebral body of L5.

**Figure 3:**
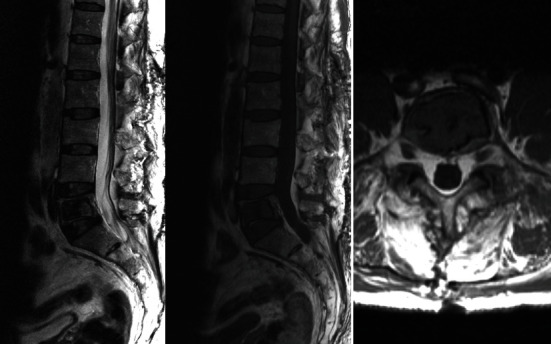
MR shows a homogeneous cystic image, hypointense in T1 sequence and hyperintense in T2, without liquid level.

The MR study showed an image of involvement of the vertebral body of L5 with a homogeneous cystic image, hypointense in T1 sequence and hyperintense in T2, without liquid level, compatible with the presence of liquid content. The rest of the spine showed no other findings except minimal lumbosacral degenerative facet changes (Figure 3).

**Figure 4:**
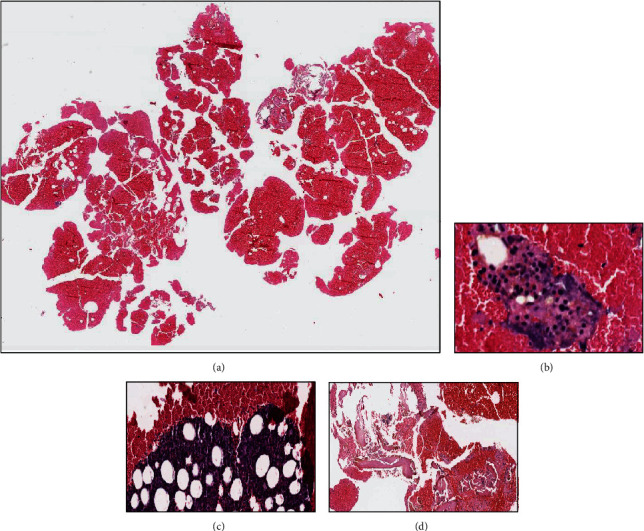
Drainage of cystic lesion. Hematoxylin and eosin staining. (a) Panoramic image of the material obtained in the drainage of the cavity, mostly hematic. (b) Hematic material with lymphocytes and isolated macrophages. (c) Lymphocyte aggregates with trapped mature adipocytes. (d) Hematic material containing small fragments of bone tissue. No osteoid material, atypical or pleomorphic cellularity, or multinucleated giant cells are observed. Neither epithelial nor endothelial cellularity is observed.

The histologic study was reported as a hematic background on which frequent neutrophils, plasma lymphocytes, and macrophages were observed, and some of them were loaded with hemosiderin and negative for malignant cells. There was no osteoid material, atypical or pleomorphic cellularity, nor multinucleated giant cells. Neither epithelial nor endothelial cellularity was observed. All this is compatible with simple bone cyst (Figure 4). PET CT was not considered since the biopsy confirmed the benign nature of the lesion. Primary malignant tumor lesions or metastases were excluded by histological study.

**Figure 5:**
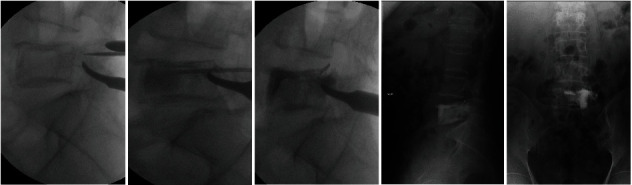
Intraoperative imaging showing PMMA injection and its irregular distribution.

With the diagnosis of the simple bone cyst and due to the size of the lesion, a second operation was decided. Under general anaesthesia, filling of the cavity with radiopaque substance (cystography) was performed through the left L5 transpedicular route in order to see the distribution of the contrast, observing during its introduction, the fall of a fragment of the cystic wall into the cavity, which was identified as “fallen leaf sign.” An attempt was made to fill the cavity with PMMA to reduce pain and prevent fracture of the vertebral body (Figure 5), and during its introduction, an irregular distribution of the contrast was observed.

Five years after the intervention, the patient is symptom-free with no pain or disability. The radiological study shows a discrete loss of lordosis, well tolerated by the patient. No changes are observed in the MR study, and the patient is active with recovery of his work activity.

## 3. Discussion

The presence of SBC in vertebral bodies and in adulthood, as in our case, is extremely infrequent. To date, only 24 cases have been described in spinal location [2] (Table 1) of which ten are located in the cervical spine [9–13, 16, 19, 22, 23, 25], two in the thoracic spine [2, 14], and thirteen in lumbar spine [2, 5–8, 15, 17, 18, 20, 21, 24, 26, 27]. In spinal localization, there is no preference for anatomical location, with eight cases described in the posterior column: spinous process [7, 9, 12–14], lamina [8], and pedicle [5, 15]. In the vertebral body, ten cases have been described [2, 6, 10, 16–19, 22, 26, 27]: three cases in the vertebral body and pedicle [2, 20, 21], one in the body and lamina [11], and one in the vertebral body and lateral mass [23].

The pathogenesis of the lesion is controversial and has been attributed to repeated microtrauma that generates haemorrhagic changes associated with a difficulty of venous return [3]. In the absence of traumatic history, the synovial debris theory [4] would explain its origin due to the intracystic presence of inflammatory mediators such as interleukin 1 (IL-1) and the good response to treatment with intralesional corticosteroids.

The diagnosis of the lesion is based on the patient's symptoms, generally spontaneous pain or because of a fracture by rupture of the cyst in long bones. The spinal location may appear in adulthood, being an incidental radiological finding, or it may manifest itself by axial pain with or without neurologic involvement due to radicular irritation that is confirmed by imaging studies. In our case, the CT study showed a unicameral lytic lesion, although with incomplete septum, without signs of cortical thinning. For an adequate visualisation of the lesion, the removal of the previous instrumentation composed of steel was needed before the MR study was performed. In addition, during the same operation, an open transpedicular aspirate was performed through the left L5 pedicle for a histologic study. After a contrast injection, a rupture of the cortical septum was observed, which was interpreted as a “fallen leaf sign” (Figure 5). Subsequently, an MR study was performed showing a normointense signal in T1 and T2 sequences.

The presence in a vertebral body of a single-chamber lytic lesion should exclude the diagnosis of other lesions such as giant cell tumor and aneurysmal bone cyst. The absence of expansive character and the location in the vertebral body without the involvement of posterior elements and the absence of fluid levels, respectively, should exclude the diagnosis. The histologic study showed the presence of hemosiderin hematic content, compatible with simple bone cyst.

Treatment should be conditioned by the benign nature of the lesion, clinical features, and possible compromise of vertebral stability due to the location and size of the lesion. In vertebral location, previous studies have shown different treatment options such as (1) aspiration and corticosteroid injection have been described for small lesions in the vertebral body, although variable success rate has been reported and several injections are usually needed for complete healing [6]; (2) simple resection [7–9], curettage [10–15], or resection with curettage [9, 11, 15] have been used for small lesions in the posterior arch in spinous apophysis, laminae, or transverse process that do not compromise segmental stability; (3) curettage and filling with autologous graft [12, 16–22] or hydroxyapatite [13] for bone growth and to prevent fractures. Although the autograft is considered a gold standard, its availability and donor site morbidity may limit its use. Hydroxyapatite has been used for cavity filling due to its osteoconductive properties, although its resorption can be slow; (4) resection with arthrodesis due to the location and possible instability after resection [5, 10, 23, 24]; and (5) use of PMMA. The decision to pursue surgical intervention in patients with simple bone cysts is a highly individualized one. An asymptomatic lesion with satisfactory maintenance of cortical thickness may require only observation whereas a lesion with precarious cortical thinning may demand surgical intervention. The PMMA filling has been described for the treatment of SBC in the calcaneus [28]. Although no case described to date in vertebral location has been treated with PMMA injection, in our case, due to the size, location of the lesion, and age of the patient, we proceeded to a reinforcement technique by filling de vertebral body with PMMA to prevent fracture of the vertebral body due to a possible collapse, thus increasing its resistance and achieving the disappearance of the pain associated with the fracture (Figure 5). In addition to the filling of the vertebral body, the high temperatures of the solidification (exothermic reaction) can give rise to thermal necrosis of the surrounding tissue, as in its use for the treatment of aneurysmal bone cyst to induce microvascular damage in the cyst wall [29].

The evolution during follow-up has been successful, without pain or increased collapse as shown by the radiological study, as well as with functional and occupational recovery.

## 4. Conclusions

The spinal localization of SBC is extremely rare. Few cases have been described in this location. Our case represents the first case located in the vertebral body of L5 with previous vertebral instrumentation and treated with a PMMA injection.

## Figures and Tables

**Table 1:** Summary of cases.

Case	Sex	Age	Level	Location	Symptoms	Treatment	FU	Author	Year
1	M	37	C4	Body	Back pain	C&BG	10 months	Dawson	1976
2	M	13	L3	Spinous process	Back pain	Resection	1 year	Wu	1981
3	M	31	L1	Body	Back pain	C&BG	3 years	Brodsky	1986
4	M	40	L2	Body	Back pain	C&BG	7 years	Matsumoto	1990
5	F	63	C5	Body	Shoulder pain	Curettage	13 months	Nakagawa	1994
6	F	12	C2	Body and lamina	Neck pain	Curettage	2 years	Park	1997
7	M	4	C2	Body	Neck pain	C&BG	2.5 years	Shen	1998
8	M	14	C7	Spinous process	—	Curettage	10 months	Lee	2000
9	F	13	C7	Spinous process	Neck pain	Curetagge and HA	—	Zemmyo	2000
10	F	10	C7	Body and lateral mass	Neck pain	Resection and PF	1 year	Snell	2001
11	M	25	L5	Lamina	Back pain	Resection	12 months	Chang	2001
12	F	17	T9	Spinous process	Back pain	Curettage	8 months	Tsirikos	2002
13	F	27	L2	Body and pedicle	—	C&BG	—	Fujimoto	2002
14	F	7	L3	Body and pedicle	Back pain	C&BG	3 years	Amrani	2002
15	F	53	L1	Pedicle	Back pain	Resection and PF	3 years	Ha	2003
16	F	50	L3	Pedicle	Back pain	Curettage	—	Ogata	2004
17	F	22	C4	Spinous process	Neck pain	Resection	—	Coskun	2004
18	M	34	L3	Body	Back pain	—	—	Nayman	2015
19	F	28	L5	Body	Back pain	—	—	Fazeli	2016
19	F	13	C2	Body	Back pain	C&BG	12 months	Boude	2017
20	F	16	L4	Body	Back pain	Steroid injection	7	Funayama	2017
21	F	17	L4	All columns	—	Resection and PF	months 10 years	Kao	2020
22	M	24	T12	Body and pedicle	Back pain	C&BG	10 years	Safaei	2021
23	M	26	L5	Body	Back pain	C&BG	7 years	Safaei	2021
24	M	50	L5	Body	Back pain	PMMA injection	5 years	Current	2023

C&BG: curettage and bone graft; HA: hydroxyapatite; PF: posterior fusion.

## Data Availability

Our work has been based on the description and literature review of the bone cyst in the lumbar spine. The information we provide is based on the description of a clinical case from our institution as well as the review of this pathology through all the consulted bibliographies available in the manuscript.
